# Peer Mentor Training and Supervision for a Digital Adolescent Depression Treatment in South Africa and Uganda: Mixed Methods Evaluation

**DOI:** 10.2196/86470

**Published:** 2026-04-09

**Authors:** Zamakhanya Makhanya, Bianca Moffett, Julia R Pozuelo, Meghan Davis, Joy Louise Gumikiriza-Onoria, Shayni Geffen, Tlangelani Baloyi, Tholene Sodi, Eugene Kinyanda, Michelle G Craske, Christine Tusiime, Crick Lund, Alastair Van Heerden, Kathleen Kahn, Alan Stein, Heather O'Mahen, DoBAt and Ebikolwa Consortium, Alan Stein

**Affiliations:** 1 Department of Social Policy and Intervention, University of Oxford, Barnett House, 32-37 Wellington Square, Oxford, OX1 2ER, United Kingdom, 44 07470762759; 2 SAMRC/Wits Rural Public Health and Health Transitions Research Unit (Agincourt), School of Public Health, Faculty of Health Sciences, University of the Witwatersrand, Johannesburg, South Africa; 3 Centre for Social Science Research, University of Cape Town, Cape Town, South Africa; 4 Department of Global Health and Social Medicine, Harvard Medical School, Boston, MA, United States; 5 Department of Psychiatry, University of Oxford, Oxford, United Kingdom; 6 Mind Ease, London, United Kingdom; 7 Department of Psychiatry, Makerere University, Kampala, Uganda; 8 SA Federation for Mental Health, Cape Town, South Africa; 9 The Vaccines and Infectious Diseases Analytics (VIDA) Research Unit, University of the Witwatersrand, Johannesburg, South Africa; 10 SAMRC-DSI/NRF-UL SARChI Chair in Mental Health and Society, University of Limpopo, Polokwane, South Africa; 11 Department of Psychology, University of California, Los Angeles, Los Angeles, CA, United States; 12 Department of Psychiatry and Biobehavioral Sciences, University of California, Los Angeles, Los Angeles, CA, United States; 13 Department of Psychology, Butabika Hospital, Kampala, Uganda; 14 Centre for Global Mental Health, Health Service and Population Research Department, King's College London, London, United Kingdom; 15 Department of Psychiatry and Mental Health, Alan J Flisher Centre for Public Mental Health, University of Cape Town, Cape Town, South Africa; 16 SAMRC/Wits Developmental Pathways for Health Research Unit, Department of Paediatrics, School of Clinical Medicine, Faculty of Health Sciences, University of the Witwatersrand, Johannesburg, South Africa; 17 Umeå Centre for Global Health Research, Department of Public Health and Clinical Medicine, Division of Epidemiology and Global Health, Umeå University, Umeå, Sweden; 18 Africa Health Research Institute, KwaZulu Natal, South Africa; 19 Blavatnik School of Government, University of Oxford, Oxford, United Kingdom; 20 Mood Disorders Centre, Department of Psychology, University of Exeter, Exeter, United Kingdom; 21 See Acknowledgments

**Keywords:** depression, adolescent, digital interventions, mobile app, behavioral activation, program evaluation, peer support, task-sharing, training and supervision, fidelity, competence and adherence, low- and middle-income countries

## Abstract

**Background:**

Blended digital mental health interventions combining technology with human support are more effective than stand-alone treatments. However, limited research has examined how to train and supervise personnel delivering human support components. The Kuamsha app, a gamified digital intervention for adolescent depression based on behavioral activation, was designed to be paired with low-intensity telephone-based peer support. A structured training and supervision program for peer supporters was codeveloped through workshops with mental health professionals and youth with lived experience of mental health challenges in South Africa and Uganda. To the best of our knowledge, this is the first study to evaluate a structured peer mentor model within a digital mental health intervention in low- and middle-income countries.

**Objective:**

This study assessed the feasibility, acceptability, and fidelity of a training and supervision program for peer supporters delivering a digital mental health intervention in South Africa and Uganda.

**Methods:**

We conducted a mixed methods evaluation of the peer mentor program. Quantitative metrics assessed the feasibility of recruitment, retention, and attendance among peer mentors (n=13, South Africa; n=4, Uganda), as well as training acceptability. Fidelity, adherence, and competence were scored at the session level and converted to percentages of the maximum possible score. Linear mixed-effects regression models with a random intercept for provider and site estimated adjusted marginal means (95% CI). In-depth interviews and focus group discussions explored program acceptability and implementation factors.

**Results:**

The peer mentor training and supervision program was feasible and acceptable in both settings, with high recruitment (South Africa: n=13/19, 68%; Uganda: 4/4, 100%), retention (South Africa: 9/13, 69%; Uganda: 4/4, 100%), and training attendance rates (89%‐92% in South Africa and 100% in Uganda), alongside qualitative reports of high satisfaction. All peer mentors met a minimum posttraining competency threshold (≥50%), with median competency scores of 70.7% (IQR 45.8%‐78.2%) in South Africa and 75.4% (IQR 73.8%‐77.3%) in Uganda. Independent ratings of recorded calls indicated high overall fidelity in South Africa (84.7%, 95% CI 80.3%‐89.0%) and Uganda (87.7%, 95% CI 83.4%‐92.1%). Adherence was higher in Uganda than South Africa (adjusted mean difference [AMD] 13.30 percentage points, 95% CI 8.99‐17.61; *P*<.001), as was competence (AMD 4.88 percentage points, 95% CI 1.23‐8.53; *P*=.009). The AMD in overall fidelity (3.06 percentage points, 95% CI −0.98 to 7.10) was not statistically significant (*P*=.14). The qualitative findings emphasized the value of ongoing supervision and capacity development, interactive training approaches, and blended delivery models.

**Conclusions:**

Locally adapted training and supervision models can strengthen peer mentor capabilities to support digital interventions. Adequate supervisory capacity and incentive structures are critical to sustain engagement, retention, and fidelity. In settings with frequent network disruptions, periodic in-person contact between peer mentors and supervisors may enhance fidelity. Future research should examine how peer mentor fidelity influences user engagement and mental health outcomes.

## Introduction

### Burden and Unmet Need in Low- and Middle-Income Countries

Adolescent depression is a significant global public health concern and a leading contributor to disability among young people worldwide [1,2]. It is consistently linked to long-term psychosocial difficulties in adulthood, including persistent mental health issues, lower academic achievement, impaired social functioning, and unstable employment [3,4]. These challenges are particularly acute in low- and middle-income countries (LMICs), where recent studies from South Africa and Uganda estimate that up to 20% of adolescents experience depressive disorders [5-7]. In these settings, adolescent depression is also associated with substance use, sexual risk behaviors, and entrenched poverty and inequality, further exacerbating social and economic burdens [8-10]. Although effective treatments exist [11,12], access remains limited in LMICs due to shortages of trained professionals, under-resourced health systems, and stigma [10,13]. These barriers are often more severe for adolescents than for adults because youth mental health services, policies, and resources are frequently underdeveloped, contributing to substantial unmet need [14-16].

### Promise and Limits of Digital Mental Health

Digital mental health interventions (DMHIs), such as mobile apps and web-based platforms, may offer scalable, cost-effective solutions to bridge access gaps [17-19]. By delivering therapeutic content remotely, DMHIs can help overcome workforce shortages and geographic and logistical barriers to care [20,21]. The feasibility of DMHIs in LMICs is supported by increasing access to mobile phones, including smartphones, and rising digital literacy among young people. National survey data from several Southern African countries indicate that mobile phone ownership among youth aged 15 to 24 years ranges from 32% in Malawi to 83% in Eswatini and is trending upward [22]. In South Africa, recent data from urban adolescents indicate that more than 85% report owning a smartphone and nearly 70% use the internet to seek health-related information [23]. These patterns suggest that mobile-based interventions have become a more viable strategy for reaching adolescents with mental health support. Realizing this potential, however, requires attention to equity, contextual relevance, and inclusive design, priorities that are increasingly underscored in the digital health promotion literature [24].

### Human Support and Task-Sharing

Despite this potential, digital-only interventions face important limitations, including high attrition and lower-than-expected effectiveness outcomes [19,25]. Meta-analytic evidence suggests that blended models, which combine technology with real-time communication, social connection, and accountability [26,27], improve engagement, adherence, and clinical outcomes [28-30]. Task-sharing approaches have emerged as a promising strategy to address workforce gaps in LMICs [31,32]. In these models, nonspecialists, defined as individuals without formal professional training in mental health (eg, lay counselors or community health workers), are trained and supervised to provide low-intensity psychosocial support, often alongside digital tools [21]. A newer variation within task-sharing is the peer support model. Peer supporters are nonspecialists who share similar sociodemographic characteristics (eg, age, ethnicity, or cultural background) and/or lived experience of mental health challenges with the target population [33]. Among adolescents, such shared identity may enhance trust, relatability, and engagement, potentially overcoming barriers often encountered in traditional clinical settings [34-38].

### Training, Supervision, and Fidelity

Although blended approaches have demonstrated success across diverse settings [39,40], variations in training and supervision can affect their effectiveness [41]. Central to this challenge is fidelity, defined as providers’ adherence to intervention protocols and their competence or skill in delivering the intervention [42,43]. Fidelity influences outcomes across psychological treatments in both routine clinical practice and controlled trials [44-46]. Maintaining fidelity is especially critical when interventions are delivered by nonspecialists, given their less extensive mental health training, clinical experience, and the potential impact on treatment effectiveness [20,47]. At the same time, overly rigid protocols can constrain provider competence and authentic engagement with participants, necessitating a balance between adherence and flexibility.

Structured training programs for nonspecialist health care workers and lay counselors can improve the effectiveness of psychological treatments [20,32]. Ongoing supervision supports sustained fidelity and provider competence over time [48]. However, evidence on how best to design and deliver training and supervision programs for peer-supported digital interventions remains limited, particularly in low-resource settings. The rapid expansion of digital technologies creates opportunities to provide training and supervision remotely, potentially increasing the scalability and reach of treatments [49,50]. Rigorous evaluation of the feasibility, acceptability, and outcomes of such models can inform program adaptation, implementation, and scale-up. This is particularly important in relation to fidelity and downstream intervention effectiveness.

### Study Context and Objective

Kuamsha is a gamified mobile app that delivers behavioral activation (BA) through interactive narrative stories [51]. Users choose between 2 storylines, each comprising 6 sequential modules. Each module takes approximately 15 to 20 minutes and introduces a core BA skill (further details are available in separate publications [52,53]). The intervention schedule comprised 6 modules over 11 weeks. To enhance engagement and reinforce BA concepts, trained and supervised peer mentors made brief weekly telephone calls to participants. Each adolescent was paired with a peer mentor and received up to 7 calls: 1 introductory call and 1 call per module. We reported outcomes of Kuamsha among users elsewhere [53,54].

The objective of this study was to evaluate the feasibility, acceptability, and fidelity of peer mentor training and supervision among delivery agents (ie, peer mentors) in South Africa and Uganda.

## Methods

### Study Design and Registration

This mixed methods evaluation of the peer mentor training and supervision program was embedded within 2 related feasibility studies conducted in South Africa and Uganda. In South Africa, the evaluation was nested within the digital delivery of BA therapy to overcome depression and facilitate social and economic transitions of adolescents (DoBAt) study, a 2-arm pilot randomized controlled trial assessing the feasibility, acceptability, and preliminary effectiveness of the Kuamsha app with peer support [53]. The DoBAt study was prospectively registered with the Pan African Clinical Trials Registry (PACTR202206574814636). In Uganda, the evaluation was conducted within Ebikolwa n’Empisa, a single-arm feasibility study of the Kuamsha app with peer support, adapted for a peri-urban Ugandan context [54].

This study focused specifically on evaluating the feasibility, acceptability, and fidelity of the peer mentor training and supervision model across both settings, using a convergent mixed methods design. Quantitative and qualitative data were collected and analyzed simultaneously with equal emphasis. The findings were integrated using a merging approach to provide complementary insights into feasibility, acceptability, and fidelity (see the GRAMMS [Good Reporting of A Mixed Methods Study] checklist; Checklist 1).

### Ethical Considerations

Ethical approval was obtained from institutional review boards in South Africa (University of the Witwatersrand Human Research Ethics Committee, MED20-05-01, and the Mpumalanga Province Research Committee), Uganda (Makerere University School of Public Health [HDREC750] and the Uganda National Council for Science and Technology [HS724ES]), and the United Kingdom (Oxford Tropical Research Ethics Committee, OxTREC 72‐19 and OxTREC 34‐20).

Peer mentors were provided with written information about the study objectives, procedures, potential risks and benefits, and their rights as participants, including the voluntary nature of participation and their right to withdraw at any time without consequence to their employment. Written informed consent was obtained prior to participation. Adolescents provided informed assent or consent, and parental consent was obtained for their participation and for the recording of peer mentor calls.

Confidentiality and privacy were protected throughout the study. Audio recordings and transcripts were deidentified and stored on secure, password-protected institutional servers accessible only to authorized members of the research team. Quotations included in this manuscript have been anonymized to prevent identification.

Peer mentors received reimbursement for their role in delivering the intervention, as described in Table 2; no additional compensation was provided specifically for participation in this evaluation. Risks arising during the study were managed in accordance with a predefined Risk Management Protocol [52], with supervisors conducting assessments and facilitating referrals to local services when necessary.

### Study Setting

In South Africa, the DoBAt study [53,55] took place in the Bushbuckridge subdistrict of the Mpumalanga Province, within the Health and Socio-Demographic Surveillance System study area of the South African Medical Research Council/Wits Rural Public Health and Health Transitions Research Unit (Agincourt) [56]. Bushbuckridge is a rural yet densely populated region located near the Mozambique border and is home to approximately 1 million people. Most adolescents attend secondary school, and youth literacy rates are high (91%‐95.7%); however, 36.8% of young adults (18‐30 y) are not employed, in tertiary education, or in training [57,58].

In Uganda, the Ebikolwa n’Empisa study [54] was implemented by BRAC Uganda in catchment areas mapped around the Katabi town in Wakiso District, a peri-urban region approximately 30 km from the capital, Kampala, with an estimated population of 170,000 [59]. Approximately half of adolescents attend secondary school; however, given the predominance of subsistence farming, not employed, in tertiary education, or in training rates are around 15% [60].

In both contexts, access to mental health services remains sparse, with specialist mental health care usually concentrated in district or regional hospitals. Accessing these facilities often requires lengthy travel, commonly a 2-hour journey by public transportation, and families face additional barriers due to transport costs and the opportunity costs of lost work or schooling [61,62]. The scarcity of mental health human resources further exacerbates these access challenges. South Africa has an estimated 1.3 psychiatrists and 8.5 mental health nurses per 100,000 people, while Uganda has fewer than 0.1 psychiatrists and 1.4 mental health nurses per 100,000, with most based in Kampala [62,63]. Given these constraints, we adopted a peer-mentor model to complement the Kuamsha app and extend the reach of psychological treatments to low-resource environments.

### Peer Mentor Training and Supervision

#### Development of the Peer Mentor Program

The peer mentor training and supervision program was codeveloped through a series of workshops. First, we conducted 6 online workshops with 8 mental health professionals (HOM, MGC, CL, TS, EK, AS, BM, and JLG-O) to develop a draft peer mentor manual. Next, we held in-person workshops with youth with lived experience in South Africa (4 workshops with 6‐8 participants each) and Uganda (6 workshops with 8‐10 participants each) to refine the program’s content and structure. Specialists from the United Kingdom, United States, and Uganda (HOM, AS, MGC, EK) provided expert input on BA. The training program drew from established models of peer-to-peer coaching, particularly the Screening and Treatment for Anxiety and Depression program developed by MGC at University of California, Los Angeles [39,64]. The co-design process shaped the structure of the program, which was iteratively refined to (1) build core competencies required to deliver telephone-based support to Kuamsha users and (2) optimize cultural and contextual suitability. Multimedia Appendix 1 outlines the development process.

The co-design process shaped the role specifications for peer mentors. The specifications focused on recruiting supporters close in age to, but slightly older than, adolescent users (15‐19 years), typically 18 to 30 years, as age proximity was viewed as key to relatability and shared experiences of adolescence. Additional requirements included fluency in the local language and familiarity with the local culture. Other qualities included respect for confidentiality; a basic understanding of mental health problems; and a nonjudgmental, supportive attitude. The lived experience of mental health challenges was also considered valuable for empathy and rapport; however, we did not conduct formal assessments nor require mentors to disclose personal experiences of depression due to concerns about mental health–related stigma in South Africa and Uganda.

#### Recruitment of Peer Mentors

In South Africa, we recruited applicants through advertisements circulated via university psychology and social work departments. A total of 19 Xitsonga-speaking students or recent graduates with a bachelor’s degree in psychology or social work participated in training. In Uganda, 4 youth workers (3 women and 1 man) were trained as peer mentors. Their academic backgrounds included counseling, guidance and psychology, and library and information science. All were fluent in Luganda.

#### Training Content and Format

Training was conducted online between May and November 2021, focusing on both program-specific competencies (ie, delivering BA telephone-based support) and nonspecific skills needed to establish a therapeutic alliance (Table 1). In addition to these core competencies, peer mentors received training on the study context, research ethics, and risk management. The training included didactic elements (presentations) and interactive components (skills rehearsal, role-plays) and used multimedia materials, including video content, learner workbooks, and structured call sheets.

**Table 1.** Core competencies of the peer mentor program.

Competency domain	Observable behavior or evidence
Program-specific competencies	
Behavioral activation: knowledge and skills	Explains how avoidance and withdrawal reinforce low mood Explains why identifying and engaging in personally meaningful activities can break this cycle Describes how to take small steps toward their goals Identifies strategies to overcome common barriers to engagement (sleep, interpersonal relationships, problem-solving)
Structured weekly call delivery	Introduces the call and reiterates confidentiality Inquires about the participant’s mood Assesses their engagement with the app over the past week Supports understanding and engagement with weekly activities Summarizes the call and schedules the next one
Nonspecific competencies	
Professionalism	Is prepared for each call Follows the 5 steps of the call Delivers 15‐ to 20-minute calls consistently
Ethical standards	Upholds confidentiality except in cases of risk of harm to self or others
Nonjudgmental stance	Respects differences in cultural backgrounds or belief systems Listens to participants’ ideas
Self-reflection	Reflects on performance Seeks opportunities to improve competencies
Active listening	Listens without interrupting excessively Asks open-ended questions Checks to ensure they have understood their mentee correctly

#### Supervision

Weekly online group supervision sessions (1 hour in South Africa; 30 minutes in Uganda) encouraged reflection, addressed challenges, and supported ongoing skills development. To optimize fidelity and enhance peer mentor competencies, all calls were recorded. Supervisors reviewed selected recordings and provided structured feedback to peer mentors. In both settings, supervisors were mental health professionals (a clinical psychologist in South Africa and a psychiatric clinical officer in Uganda). Supervisors conducted additional brief reflective check-ins with individual peer mentors to monitor well-being and reduce the risk of burnout.

#### Delivery of Training and Supervision

The content and materials used for training and supervision were standardized across sites; however, delivery was adapted to account for local resource availability and logistical considerations related to the study designs. For example, in South Africa, due to the wider geographical distribution of peer mentors, training was conducted entirely remotely via Microsoft Teams, with no in-person contact between peer mentors or with their trainers. In Uganda, peer mentors met at the BRAC office for online training delivered via Microsoft Teams. We have summarized the key differences in Table 2.

**Table 2.** Delivery of the peer mentor program in South Africa and Uganda.

Program characteristic	South Africa	Uganda
Peer mentors trained	19	4
Peer mentors delivering program	13	4
Employment period	Part-time, approximately 12 months	Full-time, approximately 3 months
Reimbursement	Approximately US $123 per month per mentor; mobile phone credit and data vouchers provided	US $100 per month per mentor
Training hours	36	78
Trainer-trainee ratio	1:10 (1:5 during intervention period)	1:2
Supervisor characteristics	Clinical psychologist	Psychiatric clinical officer

A TIDieR (Template for Intervention Description and Replication) checklist describing the training and supervision components (content, dose, providers, mode, tailoring, modifications, and fidelity) is provided in Checklist 2.

### Outcomes

#### Feasibility and Acceptability

Feasibility and acceptability were assessed quantitatively using supervisors’ recruitment and retention records and training and supervision attendance registers. Peer mentors also completed a structured 36-item Peer Mentor Training Evaluation questionnaire at the end of training (Multimedia Appendix 2). Focus group discussions (Multimedia Appendix 3) with peer mentors in South Africa (n=10) and Uganda (n=4) and follow-up in-depth interviews (Multimedia Appendix 4) with peer mentors in South Africa (n=9) provided further insights into satisfaction, perceived value, implementation challenges, and opportunities for improvement.

#### Fidelity

##### Posttraining Competence

We assessed peer mentors’ competence post training using a posttraining assessment developed for this study (Multimedia Appendix 5).

Several established competency frameworks guide the training and assessment of nonspecialist mental health providers, including the Enhancing Assessment of Common Therapeutic Factors and the Working With Children – Assessment of Competencies Tool, both developed within the World Health Organization’s Ensuring Quality in Psychological Support initiative [65-67]. These frameworks emphasize the assessment of core, transdiagnostic counseling competencies (eg, communication skills, ethical practice) that are applicable across psychological interventions and delivery contexts. While these tools provide a robust foundation for evaluating nonspecific competencies, we intentionally developed a program-specific assessment to capture competencies central to the peer mentor role in this intervention, including BA-specific skills (eg, activity monitoring, goal setting) and structured support tasks required to accompany a digital treatment.

##### Intervention Period Fidelity

Independent assessors evaluated peer-mentor fidelity during intervention delivery. In South Africa, approximately 10% of calls were randomly selected using proportional sampling, stratified by peer mentor, excluding introductory, incomplete, corrupted, or termination calls. In Uganda, we randomly selected 1 recording per peer mentor per week.

Recordings were evaluated using a supervisor feedback form that assessed competence (score out of 30) and adherence (scored out of 37). Fidelity scores were calculated as the sum of adherence and competence scores (Multimedia Appendix 6).

To ensure interrater reliability, 2 independent assessors double-rated 5 recordings in South Africa and 7 recordings in Uganda. The assessors resolved discrepancies through discussion and, once they reached consensus on their scoring, proceeded independently.

### Data Analysis

Quantitative data analyses were conducted using Microsoft Excel and Stata 19 SE (StataCorp LLC). Descriptive statistics summarized recruitment, retention, attendance, and implementation outcomes. Continuous variables are presented as means and SDs or medians and IQR, as appropriate, and categorical variables as counts and percentages. To compare posttraining competence scores between South Africa and Uganda, we used the Wilcoxon rank sum test.

Intervention period fidelity (0‐37), adherence (0‐7), and competence (0‐30) were scored at the session level and converted to percentages of the maximum possible score to facilitate comparison across outcomes. Because multiple sessions were delivered by the same peer mentor, observations were clustered within providers. To account for this hierarchical structure, we fitted linear mixed-effects regression models with a random provider intercept and included site (Uganda vs South Africa) as a fixed effect. Adjusted marginal means and 95% CI were estimated using postestimation margins commands, and absolute percentage-point differences between sites were derived from pairwise comparisons of marginal predictions. Intraclass correlation coefficients were calculated from model variance components to quantify provider-level clustering.

Given that only 1 implementation site was included per country, comparisons reflect site-level differences rather than country-level effects. Statistical significance was defined as *P*<.05 (2-sided).

For the qualitative component, experienced bilingual research assistants translated audio recordings from focus group discussions and in-depth interviews into English and transcribed them using a naturalized verbatim approach. Two researchers then analyzed transcripts thematically, following Braun and Clarke’s 6-phase analytical approach [68-70]: (1) familiarization with the data, (2) generating initial codes, (3) generating themes, (4) reviewing potential themes, (5) defining and naming themes, and (6) producing the report. We used NVivo 10 Software (QRS International) for data management and coding.

The qualitative analysis was informed by a contextualist epistemological position, acknowledging that participants’ accounts reflect both individual experiences and the social and implementation contexts in which the peer mentor program was delivered. Reflexivity was supported through analytic discussions among the research team, during which assumptions, positionalities, and emerging interpretations were examined and refined.

Within this broader mixed methods framework, a convergent mixed methods design was used, with quantitative and qualitative data analyzed separately and then integrated during interpretation. The findings from both data strands were compared to identify convergence, complementarity, and divergence across feasibility, acceptability, and fidelity outcomes.

## Results

### Feasibility and Acceptability

#### Recruitment and Retention Rates

In South Africa, 19 individuals who met the peer mentor person specifications participated in training. Of these, 13 (68%) peer mentor trainees achieved a competency score ≥50% (median 70.7%, IQR 45.8%‐78.2%). We recruited them to participate as peer mentors (of these trainees, 2 with borderline initial scores passed on their second attempts). During the DoBAt pilot randomized controlled trial (approximately 12 mo), 4 of 13 (31%) peer mentors withdrew from the program, resulting in a retention rate of 69%. Baseline characteristics of peer mentors who withdrew (n=4) and those who completed (n=9) are presented descriptively (see Multimedia Appendix 7). Given the small sample size and number of withdrawals, formal statistical comparisons were not conducted, as such analyses would be underpowered and potentially misleading. Those who withdrew were slightly older on average (mean 24.8, SD 3.0 y vs mean 22.7, SD 1.3 y), while distributions of gender and qualification background appeared similar across groups. These descriptive observations should be interpreted cautiously, as the small sample sizes preclude meaningful inference regarding baseline differences. Reported reasons for withdrawal included new employment or study opportunities, dissatisfaction with compensation, and declining to address performance concerns raised by supervisors.

In Uganda, 4 individuals who met the role specifications underwent training. All 4 (100%) trainees achieved a competency score ≥50% (median 75.4%, IQR 73.9%‐77.3%) and were recruited as peer mentors. During the Ebikolwa n’Empisa feasibility study (approximately 3 months), no peer mentors withdrew (100% retention).

#### Attendance Rates

Training attendance was high across both sites. In South Africa, trainees attended 6.3 (89%) out of 7 of the initial training sessions, and those selected attended 9.2 (92%) out of 10 of the booster training sessions. In Uganda, all 4 trainees attended 100% of the sessions.

Attendance at weekly supervision sessions varied. In South Africa, median attendance among mentors was 23 (IQR 21.0‐36.5) out of 40 sessions (57.5%) over 12 months. In Uganda, supervisors reported near-full attendance over the 3 months, despite no formal attendance logs being kept.

#### Peer Mentor Training Evaluation

**Figure 1.**
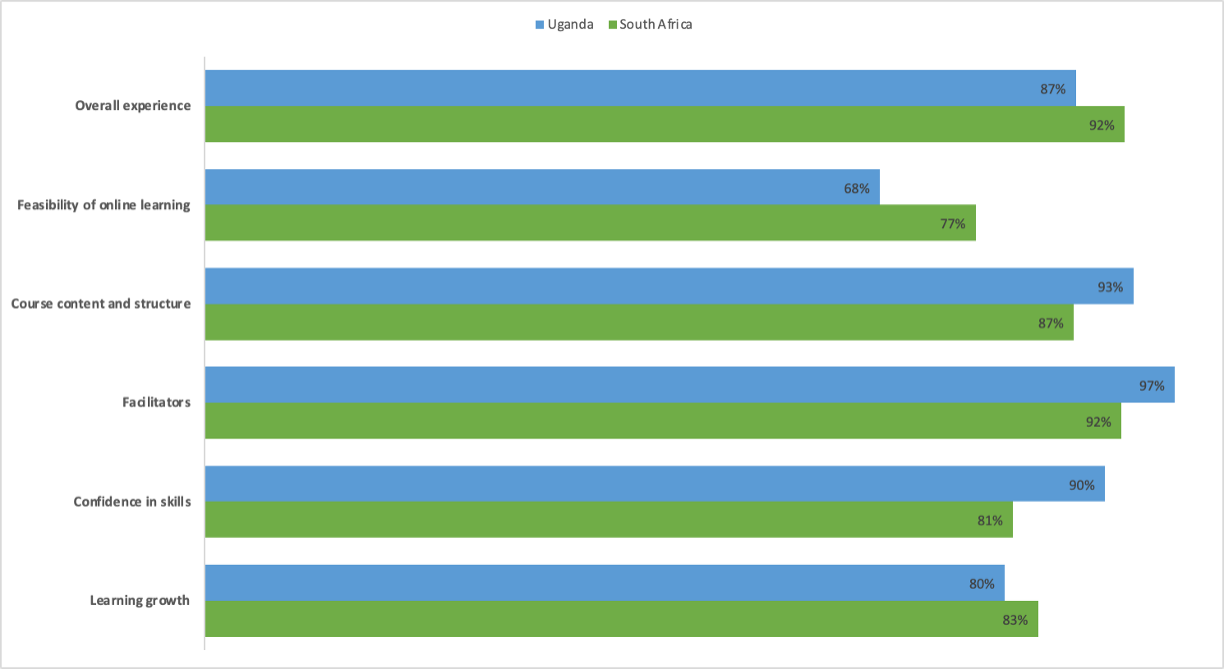
Peer mentor training evaluation scores in South Africa and Uganda.

The results indicated high overall satisfaction in both South Africa and Uganda (Figure 1). Peer mentors reported that training enhanced learning, boosted confidence in mentoring skills, and improved the understanding of BA. Facilitators were commended for clear communication and for fostering a supportive learning environment. Role-plays, instructional videos, and learner workbooks were rated as particularly useful for bridging theory and practice. While most mentors felt well prepared, a few expressed a desire for additional support to better prepare them for their roles.

One area of divergence was the feasibility of online learning, which received lower ratings than other domains. In both settings, trainees reported occasional disruptions due to power outages and network interruptions, as well as intermittent disengagement. Suggestions for improvement included scheduled breaks and adjustments to training hours.

Qualitative findings support the high acceptability of the training.

*When we did the training, I felt like, “Yes, I’m on top of this thing.*

[PM 8, focus group]

*From my side, I felt like we had a lot of training. The training was good. The content was great, and also the role-plays*.

[PM 9, focus group]

*For the first time, I was engaging in behavioral activation. Even today, in my practice, I apply it*.

[PM 6, interview]

Taken together, these findings suggest strong overall acceptability of the training model, while also identifying opportunities for refinement, particularly in settings relying on remote delivery.

### Fidelity

#### Posttraining Competence

We assessed trainees’ competence and engagement using a structured posttraining assessment; median competence was high at both sites (Table 3).

**Table 3.** Posttraining competence assessment scores.

Competency domain	South Africa (n=19), median (IQR)	Uganda (n=4), median (IQR)	*P* value^a^
Nonspecific competencies	74.3 (50.0‐83.6)	75.7 (73.6‐78.6)	.83
Program-specific competencies	75.0 (56.3‐81.7)	78.3 (72.5‐80.8)	.63
Posttraining total for group	70.7 (45.8‐78.2)	75.4 (73.8‐77.3)	.37
Overall reliability (Krippendorff *α*)	0.67	—^b^	—

a Mann-Whitney *U* test (exact *P* value).

b Not applicable.

#### Intervention Period Fidelity

A total of 89 sessions were rated (South Africa: 40 sessions across 10 providers; Uganda: 49 sessions across 4 providers). Table 4 provides adjusted marginal means for competence, adherence, and overall fidelity. Competence was 83.8% (95% CI 79.7%‐87.8%) in South Africa and 88.6% (95% CI 84.6‐92.6) in Uganda. Competence was higher in Uganda, with an adjusted mean difference of 4.88 percentage points (95% CI 1.23‐8.53; *P*=.009). Adherence was 79.7% (95% CI 75.6%‐83.7%) in South Africa and 93.0% (95% CI 89.0%‐96.9%) in Uganda, corresponding to an adjusted mean difference of 13.30 percentage points (95% CI 8.99‐17.61; *P*<.001). Overall fidelity was 84.7% (95% CI 80.3%‐89.0%) in South Africa and 87.7% (95% CI 83.4%‐92.1%) in Uganda; the adjusted mean difference of 3.06 percentage points was not statistically significant (95% CI −0.98 to 7.10; *P*=.14). Likelihood ratio tests supported the inclusion of provider-level random effects for adherence (*P*=.009), competence (*P*<.001), and fidelity (*P*<.001), indicating meaningful between-provider variability.

**Table 4.** Fidelity of peer mentor calls in South Africa and Uganda.

Outcome	South Africa (n=40 calls)	Uganda (n=49 calls)	*P* value
Competence^a^	83.8 (79.7‐87.8)	88.6 (84.6‐92.6)	.009
Adherence^a^	79.7 (75.6‐83.7)	93.0 (89.0‐96.9)	<.001
Fidelity score^a^	84.7 (80.3‐89.0)	87.7 (83.4‐92.1)	.14

a Adjusted marginal means with 95% CI.

Across outcomes, scores were generally high, with consistently higher adherence and competence in the Ugandan site. Because only 1 implementation site was included per country, these comparisons reflect site-level differences and should not be interpreted as country-level effects. Fidelity did not vary significantly across the 6 treatment calls (*F*_6,82_=1.31; *P*=.26), indicating that fidelity did not differ systematically across treatment phases. We also examined fidelity over calendar time (week of delivery) and found no significant trend (*F*_17,69_=0.76; *P*=.73), indicating that peer mentor performance remained stable throughout the study.

Qualitative perspectives on supervision highlighted barriers and facilitators to program delivery, underscoring opportunities to enhance peer mentor engagement, competence, and adherence.

Peer mentors reported that feedback on call recordings helped them develop their skills.

*That actually was very important because you could receive feedback and actually see how you’re doing and where to improve*.

[PM 2, interview]

Group supervision was considered valuable because it enabled peer mentors to learn from one another.

*If I come across a challenge, then I can say, “My mentee was difficult, and this is how I worked around it.” The next person has now taken that skill to say, “I can look out for certain things, and when that time comes, I now have something that I can use to mitigate that problem.”*

[PM 8, focus group]

For peer mentors in South Africa employed across the DoBAt pilot trial (approximately 12 months), barriers to engagement included competing work and studies.

*It was just a bit difficult for me to juggle around (my work) and be able to give all my mentees my full attention at the same time*.

[PM 4, interview]

While many South African peer mentors found the online nature of the work convenient, some felt that periodic in-person contact with supervisors and fellow mentors would have supported engagement and retention.

*I think that can be a barrier in itself, not having the in-person contact*.

[PM 2, interview]

## Discussion

### Principal Findings

This study evaluated the feasibility, acceptability, and fidelity of an online training and supervision program for peer mentors supporting the delivery of a BA digital intervention. This intervention (Kuamsha app) targeted adolescents with depression across 2 studies conducted in South Africa and Uganda. The findings suggest that the peer mentor program offered a feasible and acceptable approach in both settings and achieved high overall fidelity. Differences in retention, adherence, and fidelity across sites underscore the importance of implementation factors, such as supervision models.

In South Africa, we trained 19 peer mentors and recruited 13 for the study, with a 12-month retention rate of 9 of 13 (69%). In Uganda, all 4 trained mentors were retained in the 3-month program. Training attendance was consistently high in both countries (approximately 90% in South Africa; 100% in Uganda), but supervision attendance was more variable in South Africa (64%); in Uganda, supervisors reported few absences, although formal logs were not kept. In South Africa, weekly online group supervision followed a structured format, whereas in Uganda, supervision was more informal.

Satisfaction ratings and qualitative findings support the high overall acceptability of training and supervision. Peer mentors reported positive learning experiences, greater confidence in their acquired mentorship skills, and appreciation for multimodal and interactive training methods (eg, role-plays, guided workbooks, supervised practice). However, technical challenges, such as network disruptions and power outages, hindered participation in online training. For some peer mentors in South Africa, the part-time nature of the work and competing priorities reduced engagement in the program. Addressing these obstacles may improve retention and adherence. Examples of possible solutions include flexible online training schedules, opportunities for periodic in-person contact, and, where feasible, full-time roles with higher remuneration.

Competence assessments showed that all peer mentors met the minimum competency threshold (50%). Fidelity assessments indicated moderate-to-high adherence in both countries and remained stable across treatment phases and over time. These findings align with prior research demonstrating that digital interventions are feasible and can provide effective mental health support in LMICs [19]. Competence and adherence were higher in the Ugandan than the South African site, which is unsurprising given the shorter program duration (3 months as opposed to 12 months) and higher supervisor: peer mentor ratio (1:4, as opposed to 1:13). It is also possible that some in-person contact of peer mentors with their supervisors in Uganda contributed to engagement, retention, and performance. These findings highlight the need for adequate supervisory capacity to maintain fidelity.

### Comparison With Prior Work

The findings of this study contribute to the broader literature on training nonspecialist providers to deliver mental health interventions in LMICs and add emerging evidence on training and supervising peer supporters. A recent systematic umbrella review found that peer-delivered interventions are more likely to succeed when peer supporters receive structured training, ongoing supervision, and clear role guidance. However, most of this evidence comes from high-income settings [71]. The DoBAt pilot trial findings indicate that peer mentors promoted engagement and adherence to the app, as evidenced by a positive association between call frequency and usage metrics, as well as adolescents’ reports of the value of peer guidance [53].

Research on DMHIs suggests that human-supported approaches tend to yield better adherence than digital-only interventions. Our study contributes to this evidence base by demonstrating that structured online supervision and booster training can help maintain fidelity in peer-led interventions in low-resource settings [72]. The differences observed between South Africa and Uganda mirror previous task-sharing research, which suggests that economic and contextual factors influence volunteer retention and performance. Prior research also highlights the necessity of ongoing supervision and refresher training to maintain fidelity in peer-delivered mental health interventions [20]. Collectively, these findings support context-specific training and supervision to optimize program outcomes.

### Implications for Future Research and Practice

The results have several implications for future training and supervision models in LMICs. Retention remains a key challenge, as competing work or study priorities, or difficulty achieving competencies, may reduce long-term engagement and performance [73]. Competence assessment should remain central in peer-delivered interventions. Established tools, such as Enhancing Assessment of Common Therapeutic, Working with Children – Assessment of Competencies Tool, and the broader Ensuring Quality in Psychological Support platform, have been widely used to evaluate the clinical competencies of nonspecialist providers and share commonalities with the structured supervisor rating form used in this study [65-67]. However, our assessment was intentionally designed to capture BA-specific skills (eg, activity monitoring, structured goal setting) that are not fully encompassed in generalist tools. Future research could examine how BA-specific assessments integrate with or benchmark against existing competency frameworks.

Flexible and hybrid training and supervision models, financial incentives, career development opportunities, and credentialing or certification should be explored to improve retention. Additionally, broadening recruitment to peer mentors who meet the role requirements (eg, age, language, cultural familiarity, confidentiality, and supportive attitude) but are not limited to psychology or social work students or graduates could expand the applicant pool. Recruiting peers from local communities would also facilitate periodic in-person contact among peer mentors, mentees, and supervisors. To further reduce barriers to engagement, future programs should budget for data or mobile credit or negotiate zero-rated access, as digital inequities can disrupt call quality and limit app engagement. This recommendation is consistent with digital health scale-up frameworks that emphasize data affordability as a key barrier in LMICs [74,75].

Structured, ongoing supervision was critical for maintaining fidelity, particularly among mentors with lower adherence scores. Programs can build on these findings by optimizing remote digital and peer-led group supervision to support cost-effective and sustainable implementation [76]. Addressing logistical challenges, such as network disruptions and power outages, will further improve delivery. Access to reliable technology and alternative communication strategies may enhance engagement and prevent disruptions.

Finally, future research should evaluate clinical outcomes associated with peer mentorship in adolescent mental health because while peer-delivered and peer-supported approaches are increasingly used, the evidence base remains mixed, particularly in LMIC settings. Recent reviews of peer-led mental health interventions for youth (10‐24 y) found promising effects in several programs, but also highlighted substantial variability in intervention models (eg, structured vs informal support), outcomes, and implementation quality, alongside limited reporting on supervision, fidelity, and mechanisms through which peer support may influence symptom change [35,77]. Additionally, the outcome findings from the DoBAt pilot trial indicate that peer support may plausibly influence downstream outcomes through engagement pathways (eg, associations between mentor call frequency and intervention use). However, the pilot was not powered to isolate peer effects on depressive symptoms [53]. Together, these observations motivate fully powered studies that (1) link peer mentor fidelity and contact “dose” to user engagement metrics; (2) test whether engagement mediates symptom change; and (3) examine how modifiable implementation factors, such as supervision intensity, mentor employment conditions, and training modality, affect both fidelity and clinical outcomes.

### Strengths and Limitations

Overall, the findings highlight both promise and practical challenges of implementing peer mentor models in LMIC contexts. We have noted several strengths of this study. First, the peer mentor program was implemented and evaluated across 2 distinct low-resource settings, a rural district in South Africa and a peri-urban area in Uganda, enhancing the generalizability and relevance of the findings to diverse contexts. Second, the program was co-designed with mental health professionals and youth with lived experience, ensuring that the training content, delivery format, and support structures were grounded in clinical expertise and the realities of providers (peer mentors) and end users (adolescents). Third, the use of a mixed methods approach provided a comprehensive evaluation with quantitative data providing objective assessments of the program’s feasibility, acceptability, and fidelity, while qualitative insights contextualized peer mentors’ experiences, helping to interpret variability in outcomes. Fourth, the study assessed both the training phase and intervention delivery, offering insights into retention, supervision, and quality over time. Fifth, rigorous fidelity evaluation was conducted through structured tools and independent ratings of a proportion of recorded sessions, with a subset double-coded to ensure interrater reliability. Finally, the program incorporated adaptable training and supervision models that drew on interactive methods (eg, role-plays, guided workbooks) and targeted booster sessions, which are critical for supporting peer-delivered care in real-world settings. This study, therefore, contributes to the literature on task-sharing models and underscores the potential scalability of peer-delivered digital mental health programs [32].

Several limitations need to be acknowledged. First, the relatively small sample size, particularly in Uganda, restricts the generalizability of site-specific findings and precludes granular analyses (eg, by gender or education level). Second, self-reported measures, such as the peer mentor training evaluation survey, are susceptible to response bias. Third, variations in the recruitment of peer mentors and the delivery of training and supervision, as well as sample size and study duration, hinder direct comparisons of outcomes between South Africa and Uganda. Finally, we did not measure the direct clinical effect of peer mentorship on adolescent mental health outcomes. Future studies should evaluate the clinical effectiveness of peer mentorship within DMHIs. Prior research has shown that structured training and supervision can enhance the effectiveness of nonspecialist health workers delivering mental health care [21]. Additionally, digitally supported training and supervision may improve scalability while maintaining fidelity [78].

### Conclusions

This study demonstrates that peer support for a digital adolescent depression intervention in South Africa and Uganda is feasible and acceptable among providers and achieves moderate overall fidelity. These findings highlight the potential of DMHIs, supported by trained nonspecialists or peer mentors in LMICs. Further research should optimize training and supervision models and assess the effects of peer support on adolescent mental health outcomes.

## Supplementary material

10.2196/86470 Multimedia Appendix 1 Development of the peer mentor program in South Africa and Uganda.

10.2196/86470 Multimedia Appendix 2 Peer mentor training evaluation: domains and scoring.

10.2196/86470 Multimedia Appendix 3 Focus group discussion guide.

10.2196/86470 Multimedia Appendix 4 Interview guide.

10.2196/86470 Multimedia Appendix 5 Peer mentor competence assessment instrument.

10.2196/86470 Multimedia Appendix 6 Posttraining competence and intervention period fidelity: assessment overview.

10.2196/86470 Multimedia Appendix 7 Baseline characteristics of peer mentors by retention status.

10.2196/86470 Checklist 1 GRAMMS checklist for the peer mentor training and supervision program.

10.2196/86470 Checklist 2 TIDieR checklist for the peer mentor training and supervision program.

## Abbreviations

BA: behavioral activation
DMHI: digital mental health intervention
DoBAt: digital delivery of behavioral activation therapy
GRAMMS: Good Reporting of A Mixed Methods Study
LMIC: low- and middle-income country
SAPRIN: South African Population Research Infrastructure Network
TIDieR: Template for Intervention Description and Replication
