# Supplementary material for: Peer Mentor Training and Supervision for a Digital Adolescent Depression Treatment in South Africa and Uganda: Mixed Methods Evaluation
Source: JMIR Ment Health. 2026 Apr 9;13:e86470. doi: 10.2196/86470 (PMC13064885; doi:10.2196/86470)
Supplement: Multimedia Appendix 1 [file mental-v13-e86470-s001.docx]

### Multimedia Appendix 2. Development of the peer mentor program in South Africa and Uganda.

| Development Stage | Location | Activities |
| --- | --- | --- |
| **Stage 1** |  |  |
| Conceptualisation | Online | - Observation of 6 training sessions and review of training and supervision materials from the UCLA Screening and Treatment for Anxiety and Depression (STAND) program*.* - Six online workshops with researchers and local mental health professionals to outline key aspects of the peer mentor training manual, supervisor’s manual, and peer mentor person specification. - Weekly meetings with a working group of experts to draft and refine the peer mentor manual, supervisor’s manual, training and supervision materials, competency assessment, and fidelity checklist. |
| **Stage 2** |  |  |
| Stakeholder consultation | South Africa and Uganda | - Ten workshops with adolescents with lived experience, community stakeholders (caregivers of adolescents, teachers, and local youth council members), and mental health professionals to obtain feedback on the role of the peer mentor, person specification, safeguarding procedures, and community mental health resources. |
| **Stage 3** |  |  |
| Implementation | Uganda | - Four peer mentors were trained and supervised by a psychiatric clinical officer (online) with input (online) from a research assistant and clinical psychologist. |
|  | South Africa | - Nineteen peer mentors were trained, and 13 were selected to participate. Training and supervision conducted remotely (online). Two research assistants performed the training. Supervision was provided by a clinical psychologist, with input from a registered counsellor and a research assistant. |
|  | Uganda and South Africa | - Peer mentors delivered seven phone calls to participants over 11 weeks. - Peer mentors participated in weekly supervision sessions (online in SA and Uganda). - The peer mentor component was iteratively adapted during the initial implementation stage to address initial barriers. |
| **Stage 4** |  |  |
| Evaluation and further adaptation | South Africa | - A mixed-methods evaluation of the feasibility, acceptability, and fidelity of the peer mentor program was nested within a pilot randomized controlled trial to assess the Kuamsha program (N=195). - Peer mentor focus group discussions (n=10) and in-depth interviews (N=9) on barriers/facilitators to program implementation to inform further adaptation. |
|  | Uganda | - Peer mentor program evaluation nested within a single-arm feasibility and acceptability study (N=31) - Peer mentor focus group discussions (n=4) on barriers/facilitators to program implementation. |
