# Supplementary material for: Peer Mentor Training and Supervision for a Digital Adolescent Depression Treatment in South Africa and Uganda: Mixed Methods Evaluation
Source: JMIR Ment Health. 2026 Apr 9;13:e86470. doi: 10.2196/86470 (PMC13064885; doi:10.2196/86470)
Supplement: Multimedia Appendix 2 [file mental-v13-e86470-s002.docx]

### Multimedia Appendix 4. Peer mentor training evaluation: Domains and scoring.

| **Domain** | **Scoring range** | **Description/examples** |
| --- | --- | --- |
| Learning growth | 0–2 | Self-rated change in knowledge and skills from before to after training; composite improvement score. |
| Confidence in skills | 0–16 | Confidence applying core mentoring skills (eg, conducting structured calls, active listening, problem-solving). Includes one reverse-coded item.^a^ |
| Facilitators | 0–20 | Satisfaction with facilitator communication, responsiveness, and support during training. |
| Course content and structure | 0–28 | Perceived clarity, organization, workload appropriateness, and usefulness of training content and structure. |
| Feasibility of remote delivery | 0–16 | Perceptions of technical and logistical feasibility (eg, internet stability, power interruptions, platform usability). Includes one reverse-coded item.^b^ |
| Overall experience | 5–50 | Overall training experience rated on a 10‑point scale across multiple items. |

^a^ One reverse-coded item in the Confidence in skills domain.

^b^ One reverse-coded item in the “feasibility of remote delivery” domain.

**Scoring:** Most items used a Likert scale (0–4: poor to excellent or strongly disagree to strongly agree). A total score was calculated by summing all domains and expressed as a percentage (0–100), with higher scores indicating greater feasibility and acceptability.
