# Supplementary material for: Peer Mentor Training and Supervision for a Digital Adolescent Depression Treatment in South Africa and Uganda: Mixed Methods Evaluation
Source: JMIR Ment Health. 2026 Apr 9;13:e86470. doi: 10.2196/86470 (PMC13064885; doi:10.2196/86470)
Supplement: Multimedia Appendix 3 [file mental-v13-e86470-s003.docx]

### Multimedia Appendix 5. Focus group discussion guide.

**Introduction & Consent**

- Welcome participants; check recording.
- Explain purpose of the FGD:
  - To reflect on peer mentors’ experiences of participating in the Kuamsha program.
  - To gather feedback on feasibility, acceptability, training, and implementation challenges.
  - To identify areas for improvement for potential scale-up.
- Emphasize confidentiality and that there are no right or wrong answers.
- Confirm informed consent (including audio recording).
- Remind participants that the discussion will cover several sections, starting with overall impressions of the program, then training, language, and other components.
- Appoint a notetaker

**Section 1: Overall Experience with the Kuamsha Program**

**Opening prompt:**

- What was it like for you participating in the Kuamsha program over the past year?

**Follow-up probes:**

- Individual reflections (asked one by one).
  - Probe on **impact**: “Do you think the program made a difference in your mentees’ lives?”
  - Probe on **engagement**: experiences of mentees who were reachable vs those challenging to reach.
- Explore emotional connection to mentees (e.g., attachment, wondering about real-life impact)
- Discuss scheduling challenges and feasibility issues (e.g., mentees not answering calls, irregular contact)
- Explore app and dashboard functionality: experiences with Kuamsha app use, dashboard data vs mentee self-reports

**Section 2: Challenges and Facilitators**

**Prompt:**

- Can each of you share one thing that you found difficult about the program, and one thing that was easier?

**Probes:**

- Scheduling difficulties (mentees not responding, inconsistent call times).
- App technical issues (logouts, syncing problems, dashboard discrepancies).
- Reporting challenges (e.g., mood rating forms not updating).
- Problem-solving strategies peer mentors used to address these challenges.

**Section 3: Training Experiences**

**Prompt:**

- Think back to the training you received last year. How well prepared did it make you feel for the peer mentor role?

**Probes:**

- Overall impressions of content, length, and structure.
- Which parts worked well (e.g., role plays, behavior activation content)?
- Which areas could have been improved or expanded (e.g., more crisis scenarios, language-specific materials)?
- Views on whether having hands-on access to the app during training would have helped
- Discussion on use of personal vs study-provided devices for app training and delivery.
- Were there any skills you felt were missing or could have been added?

**Section 4: Language and Translation**

**Prompt:**

- Let’s talk about language. You were trained and supervised in English, but you delivered sessions in Xitsonga. How did you experience switching between the two languages?”

**Probes:**

- Challenges translating technical/psychological concepts into Xitsonga.
- Code-switching during calls and preparation needed.
- Experiences with app language (English → Xitsonga translation quality).
- Suggestions for making language more accessible for adolescents.
- Potential solutions (e.g., local teachers reviewing translations, Xitsonga call sheets, involving local developers)

**Section 5: Reflections and Improvement Suggestions**

**Prompt:**

- Thinking about everything we’ve discussed, what changes would you suggest to improve the program if it were to be scaled up?

**Probes:**

- Adjustments to app (e.g., language simplification, locking content to improve monitoring).
- Supervision or training modifications.
- Suggestions regarding language adaptation, scheduling systems, or technological support.
- Additional support peer mentors would find helpful (e.g., refresher trainings, more regular role plays, monthly training sessions)

**Closing**

- Thank participants for their honest contributions.
- Summarize key themes.
- Emphasize that their feedback will directly inform improvements in future program iterations and potential scale-up.
