# Supplementary material for: Peer Mentor Training and Supervision for a Digital Adolescent Depression Treatment in South Africa and Uganda: Mixed Methods Evaluation
Source: JMIR Ment Health. 2026 Apr 9;13:e86470. doi: 10.2196/86470 (PMC13064885; doi:10.2196/86470)
Supplement: Multimedia Appendix 4 [file mental-v13-e86470-s004.docx]

### Multimedia Appendix 6. Interview guide.

**Introduction & Consent**

- Thank the participant for their time and explain the purpose of the interview:
  - To understand their experience providing the intervention to adolescents in the DoBAt study.
  - To explore their thoughts regarding solutions to address depression and anxiety in the community. Emphasize anonymity and that there are no right or wrong answers.
- Confirm informed consent (including audio recording).
- Offer opportunity to ask questions before starting.

**Section 1: Overall Experience & Role**

**For Peer Mentors:**

- Can you tell me a little bit about your role as a peer mentor and your overall experience participating in the study?
- How many participants were you supporting during the study?
- How was that number for you? Could you have handled more, or would fewer have been better?

**For Peer Mentor Supervisors:**

- Can you tell me about your role as a peer mentor supervisor and what that involved day-to-day?”
- How did you find supervising the peer mentors and being part of the program?

**Section 2: Training and Supervision**

- Can you tell me about the training you received before starting the role? What worked well, and what challenges did you face?
- Do you feel the training prepared you for the role? If so, why?
- How did you find the amount and structure of the training (e.g., length, timing)?
- What was your experience of the supervision you received during the intervention?
  - **Probe:** group vs individual supervision preferences.
  - **Probe:** how supervision was used to address problems.

**For supervisors:**

- What did supervision sessions with peer mentors typically involve?
- How frequently did you meet, and what kinds of issues came up?

**Section 3: Barriers and Facilitators to Delivering the Intervention**

- Were there any challenges or barriers you faced when delivering the intervention?
  - **Probe:** issues with reachability of adolescents, scheduling, technology, load-shedding, and engagement.
- How did you address these challenges? (e.g., using fieldworkers, rescheduling, supervision)
- Were there any facilitators or things that made your work easier?
  - **Probe:** WhatsApp groups, support from study team, clear protocols.

**Section 4: Perceived Impact & Benefits**

- Did you notice any benefits of the intervention for the adolescents you were mentoring?
  - **Probe:** emotional well-being, problem-solving, engagement with the app, schoolwork, motivation.
- How did that make you feel as a peer mentor?

**For supervisors:**

- What changes or impacts did you observe through the supervision process (e.g., mentors’ skills, participant outcomes)?

**Section 5: Reflections and Suggestions**

- Overall, what do you think worked well in the intervention?
- Are there any aspects you would change or improve if we were to conduct the intervention again?
  - **Probe:** digital vs face-to-face delivery, app usability, supervision frequency, training content.
- Any final thoughts or reflections on your experience as a peer mentor/supervisor?

**Closing**

- Thank the participant for their time and contributions.
- Reiterate the value of their feedback for improving future interventions.
