# Supplementary material for: Peer Mentor Training and Supervision for a Digital Adolescent Depression Treatment in South Africa and Uganda: Mixed Methods Evaluation
Source: JMIR Ment Health. 2026 Apr 9;13:e86470. doi: 10.2196/86470 (PMC13064885; doi:10.2196/86470)
Supplement: Multimedia Appendix 5 [file mental-v13-e86470-s005.docx]

### Multimedia Appendix 7. Peer mentor competence assessment instrument.

| PEER MENTOR COMPETENCE ASSESSMENT | | | | | | |
| --- | --- | --- | --- | --- | --- | --- |
| Peer mentor name:  Assessor name:  Date: | | | | | | |
| SECTION A: ROLE PLAY | | | | | | |
| Program-specific competencies | **Assessment** | | | | | **Comments** |
|  | **1** | **2** | **3** | **4** | **5** |  |
| Follows Steps 1 (introduction) |  |  |  |  |  |  |
| Follows Steps 2 (how they are) |  |  |  |  |  |  |
| Follows Steps 3 (use of app) |  |  |  |  |  |  |
| Follows Steps 4 (BA activity) |  |  |  |  |  |  |
| Follows Steps 5 (summarizing) |  |  |  |  |  |  |
| Nonspecific competencies |  |  |  |  |  |  |
| Clearly explains key lessons and activities, demonstrating knowledge of the BA skills (BA Content) |  |  |  |  |  |  |
| Helps mentees to troubleshoot the weekly activities, including referring to the goal (BA Content) |  |  |  |  |  |  |
| Provides feedback/responses to mentees, using the peer mentor competency of engaging participants |  |  |  |  |  |  |
| Provides feedback/responses to mentees, using the peer mentor competency of professionalism |  |  |  |  |  |  |
| Provides feedback/responses to mentees, using the peer mentor competency of respecting individual and cultural differences |  |  |  |  |  |  |
| Provides feedback/responses to mentees, using the peer mentor competency of ethical standards |  |  |  |  |  |  |
| Provides feedback/responses to mentees, using the peer mentor competency of self-reflection |  |  |  |  |  |  |
| Completes the phone call in 15-20 minutes |  |  |  |  |  |  |
| KEY  1 Needs development (1 pt)  2 Approaching competence (2 pts)  3 Competent (3 pts)  4 Highly Competent (4 pts)  5 Exceptional (5 pts) | **TOTAL POINTS:** | | | | | |
|  | **Overall assessment:** | | | | | |

**Abbreviations:** BA, Behavioral Activation.
