# Supplementary material for: Peer Mentor Training and Supervision for a Digital Adolescent Depression Treatment in South Africa and Uganda: Mixed Methods Evaluation
Source: JMIR Ment Health. 2026 Apr 9;13:e86470. doi: 10.2196/86470 (PMC13064885; doi:10.2196/86470)
Supplement: Multimedia Appendix 6 [file mental-v13-e86470-s006.docx]

### Multimedia Appendix 8. Post-training competence and intervention-period fidelity: Assessment overview.

| **Assessment** | **How assessed** | **Raters and sampling** | **Scoring and thresholds** |
| --- | --- | --- | --- |
| Post-training competence |  |  |  |
| South Africa | Structured role-play using the Post-training Competence Assessment to evaluate BA-specific skills and nonspecific counseling skills; engagement in training assessed via short, structured items after the role-play. | Raters: clinical psychologist, research assistant, master’s student. Sample: 19 trainees assessed immediately after training. | Competence components: nonspecific competencies (max 35) + program-specific (BA) competencies (max 30) = total competence (max 65). Competency threshold: ≥50% of component maximums. |
| Uganda | Structured role-play using the same assessment as South Africa; engagement in training assessed post–role play. | Raters: psychiatric clinical officer, research assistant. Sample: 4 trainees assessed immediately after training. | Competence components identical to South Africa. Competency threshold: ≥50% of component maximums. |
| Intervention-period fidelity |  |  |  |
| South Africa | Independent rating of recorded support calls using a supervisor feedback form, capturing adherence and competence during delivery. | Raters: master’s-level research assistant and clinical psychologist. Sampling: approximately 10% of calls randomly selected per peer mentor^a^, excluding introductory, incomplete/corrupted, or termination calls. Sample size: 40 of 421 calls. | Adherence checklist (max 7) + competence rating (max 30) = fidelity score (max 37). Minimum adherence and competence thresholds: ≥50%. |
| Uganda | Independent rating of recorded support calls using the same supervisor feedback form. | Raters: two clinical psychologists. Sampling: one recording per peer mentor per week^b^, yielding ~33% coverage. Sample size: 49 of 148 calls. | Scoring identical to South Africa: adherence (max 7), competence (max 30), fidelity (max 37). Minimum thresholds: ≥50%. |

**^a^** Approximately 10% random sampling per peer mentor; introductory, incomplete/corrupted, and termination calls excluded (South Africa).
**^b^** One recording per peer mentor per week (Uganda).

**Abbreviations:** BA, Behavioral Activation
