# Supplementary material for: Peer Mentor Training and Supervision for a Digital Adolescent Depression Treatment in South Africa and Uganda: Mixed Methods Evaluation
Source: JMIR Ment Health. 2026 Apr 9;13:e86470. doi: 10.2196/86470 (PMC13064885; doi:10.2196/86470)
Supplement: Multimedia Appendix 7 [file mental-v13-e86470-s007.docx]

### Multimedia Appendix 9. Baseline characteristics of peer mentors by retention status.

| Characteristic | Remained (n=9) | Dropped Out (n=4) |
| --- | --- | --- |
| Age, mean (SD) | 22.7 (1.3) | 24.8 (3.0) |
| Female, n (%) | 6 (67) | 2 (50) |
| Male, n (%) | 3 (33) | 2 (50) |
| Qualification, n (%) |  |  |
| Social Work background | 6 (67) | 3 (75) |
| Psychology background | 3 (33) | 1 (25) |
