# Supplementary material for: Peer Mentor Training and Supervision for a Digital Adolescent Depression Treatment in South Africa and Uganda: Mixed Methods Evaluation
Source: JMIR Ment Health. 2026 Apr 9;13:e86470. doi: 10.2196/86470 (PMC13064885; doi:10.2196/86470)
Supplement: Checklist 1 [file mental-v13-e86470-s008.docx]

### Multimedia Appendix 1. GRAMMS (Good Reporting of A Mixed Methods Study) checklist for the peer mentor training and supervision program.

| Item No. | GRAMMS Criterion | Guide Question / Description | Reported Information | Section and page number |
| --- | --- | --- | --- | --- |
| 1 | Rationale for using a mixed methods design | Explain why a mixed-methods approach was chosen. | A mixed-methods approach enabled a comprehensive evaluation of feasibility, acceptability, and fidelity. Quantitative data captured recruitment, retention, attendance, and fidelity metrics, while qualitative data contextualized peer mentors’ experiences and implementation barriers/facilitators, enriching the interpretation of the numerical patterns. | Methods: Study design and registration (pages 3–4) |
| 2 | Description of the design in terms of purpose, priority, and sequence of methods | Describe the type of mixed methods design and how the qualitative and quantitative components relate. | We employed a convergent mixed-methods design. Quantitative and qualitative data were collected and analyzed in parallel with equal priority. We merged the findings at the interpretation stage to provide complementary perspectives on feasibility, acceptability, and fidelity. | Methods: Study design and registration (pages 3–4) |
| 3 | Description of each method used and where integration occurred | Detail data collection and analysis methods, and points of integration. | Quantitative data (recruitment, retention, attendance, post-training competence, intervention-period fidelity) were summarized using descriptive statistics and nonparametric tests in Stata. Qualitative data (Focus groups, interviews) were analyzed via reflexive thematic analysis (Braun & Clarke) using NVivo. Integration occurred at the interpretation stage, where qualitative themes were used to explain quantitative patterns (e.g., differences in retention and fidelity). | Methods: Outcomes; Data Analysis (pages 7–8) |
| 4 | Justification for how integration was achieved | Explain the approach to combining results (merging, connecting, or embedding). | We used a merging approach to integration: quantitative and qualitative results were brought together in the discussion section, triangulating feasibility/acceptability/fidelity and identifying contextual influences (e.g., supervision structure, remote delivery, remuneration). | Methods: Study design and registration (pages 3–4)  Discussion: Principal findings (page 12) |
| 5 | Limitations of the mixed methods design | Discuss limitations specific to using mixed methods. | Integration was interpretive rather than statistical; different sample sizes and durations constrained cross-site comparability, and qualitative findings reflect peer mentors’ perspectives. These factors may limit the extent to which integrated inferences generalize across settings. | Discussion: Strengths and limitations (page 14) |
| 6 | Insights gained from integrating methods | Describe how combining methods yielded new insights. | Integration revealed that supervision structure, delivery modality, and remuneration/employment arrangements influenced retention and fidelity—insights not apparent from quantitative data alone. Qualitative findings contextualized numerical patterns and informed recommendations for optimizing peer mentor programs in LMIC settings. | Discussion: Principal findings; Implications for future research and practice (pages 12–13) |
