# Supplementary material for: Peer Mentor Training and Supervision for a Digital Adolescent Depression Treatment in South Africa and Uganda: Mixed Methods Evaluation
Source: JMIR Ment Health. 2026 Apr 9;13:e86470. doi: 10.2196/86470 (PMC13064885; doi:10.2196/86470)
Supplement: Checklist 2 [file mental-v13-e86470-s009.docx]

### Multimedia Appendix 3. TIDieR (Template for Intervention Description and Replication) checklist for the peer mentor training and supervision program.

| Item No. | TIDieR Item | Guide Question / Description | Reported Information | Section and page number |
| --- | --- | --- | --- | --- |
| 1 | Brief Name | What is the intervention called? | Peer Mentor Training and Supervision in Adolescent Digital Treatment | N/A |
| 2 | Why (Rationale, theory, or goal) | Describe the rationale, underlying theory, or goals. | The intervention aimed to equip young peer mentors (aged 18–30) to provide low-intensity telephone-based support alongside a gamified Behavioral Activation (BA) app for adolescent depression. It was grounded in task-sharing and BA theory, and designed to enhance engagement, adherence, and delivery fidelity in digital mental health interventions in LMICs. | Introduction (pages 2–3)  Methods: Development of the peer-mentor program (page 5) |
| 3 | What (Materials) | Describe physical or informational materials used. | Training manuals, learner workbooks, structured call sheets, slide decks, video examples, and fidelity rating forms. | Methods: Training content and format (page 5)  Multimedia Appendices 4, 7, and 8 |
| 4 | What (Procedures) | Describe procedures, activities, or processes used in the intervention. | Training included didactic sessions, interactive role-plays, and guided practice focused on BA concepts, structured call delivery, research ethics/confidentiality, and reflective practice. Supervision included weekly 30-minute to 1-hour group sessions reviewing call recordings and providing structured feedback. | Methods: Training content and format; Supervision (pages 5–6) |
| 5 | Who provided | For each category of intervention provider, describe their expertise, background, and training. | Trainers: Clinical psychologists, registered counsellors, and master’s-level research assistants. Supervisors: Clinical psychologist (SA) and psychiatric clinical officer (UG). Peer mentors: young adults recruited locally (SA: psychology/social work students/graduates; UG: community youth workers) trained in BA principles. | Methods: Development; Recruitment of peer mentors; Supervision (pages 5–6) |
| 6 | How (Delivery mode) | Describe how the intervention was delivered. | In South Africa: fully remote (online training and supervision via video platforms). In Uganda: delivered online at BRAC offices. Peer mentors delivered weekly telephone support calls to adolescent app users. | Methods: Training content and format; Supervision; Delivery of training and supervision (pages 5–7) |
| 7 | Where | Describe the settings where the intervention occurred. | South Africa: Rural Bushbuckridge subdistrict (Mpumalanga), delivered remotely by mentors. Uganda: Peri-urban Katabi town (Wakiso District), mentors based in BRAC offices. | Methods: Study setting (page 4) |
| 8 | When and How Much | Describe the number of sessions, schedule, duration, intensity, or dose. | South Africa: 36 training hours; weekly 1-hour group supervision across ~12 months. Uganda: 78 training hours; weekly 30-minute supervision delivered online at BRAC offices over 3 months. Each peer mentor made up to 7 calls per participant (an introductory call and one per module, each lasting 15-20 minutes). | Methods: Training content and format; Supervision; Delivery of training and supervision (pages 5–7) |
| 9 | Tailoring | Was the intervention personalized, titrated, or adapted? | Core content and call structure were standardized across sites (ie, the same intervention was delivered to each participant). Delivery was adapted to the local context. Within calls, peer mentors provided minor individualized tailoring of activity goals aligned with each adolescent’s values and circumstances, consistent with BA. | Methods: Training content and format; Delivery of training and supervision (pages 5–7) |
| 10 | Modifications | If the intervention was modified during the study, describe changes and why. | No major protocol modifications were implemented. Minor scheduling adjustments addressed power/network disruptions and mentor availability, without changing core content or procedures. | Methods: Delivery of training and supervision (pages 6–7) |
| 11 | How well (Planned fidelity assessment) | Describe methods to assess adherence or fidelity. | Planned fidelity included post-training competence assessments and supervisor ratings of recorded calls during delivery, using predefined competence (1–5 per domain; total out of 30) and adherence checklists (total out of 7), with random sampling of calls (South Africa ≈10%; Uganda ≈33%) and double-rating for interrater reliability. | Methods: Outcomes (page 8)  Multimedia Appendices 7 and 8 |
| 12 | How well (Actual fidelity) | Describe the extent to which the intervention was delivered as planned. | All peer mentors met minimum competence thresholds (≥50%) post-training. During delivery, all rated calls met minimum adherence and competence criteria. Mean fidelity was moderate to high: SA = 31.05/37; UG = 33.41/37. Supervision and structured feedback maintained fidelity across treatment phases. | Results: Fidelity (pages 10–12) |
